# (*E*)-3-[4-(Dimethyl­amino)phen­yl]-1-(2-pyrid­yl)prop-2-en-1-one

**DOI:** 10.1107/S1600536809015244

**Published:** 2009-04-30

**Authors:** Songzhu Lin, Ruokun Jia, Xiaojun Zhang, Zhiwen Wang, Yanlin Yuan

**Affiliations:** a College of Chemical Engineering, Northest Dianli University, 132012 Jilin, People’s Republic of China

## Abstract

In the title mol­ecule, C_16_H_16_N_2_O, the pyridine ring and non-H atoms of the =CH—C(=O)— unit are coplaner, the largest deviation being 0.045 (2) Å for the O atom. The dihedral angle between this plane and the benzene ring is 2.79 (2)°. The mol­ecular structure is stabilized by inter­molecular C—H⋯π and inter­actions.

## Related literature

For a related structure, see: Butcher *et al.* (2007 ▶). For the phamacological activity of chalcones, see: Zhao *et al.* (2007 ▶); Fichou *et al.* (1988 ▶). For the blue-light transmittance of chalcone derivatives, see: Sarojini *et al.* (2006 ▶).
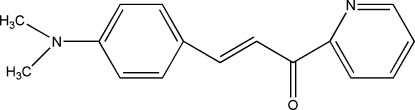


## Experimental

### 

#### Crystal data

C_16_H_16_N_2_O
*M* *_r_* = 252.31
Monoclinic, 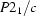

*a* = 8.1553 (4) Å
*b* = 17.4543 (12) Å
*c* = 12.1087 (5) Å
β = 125.032 (5)°
*V* = 1411.35 (16) Å^3^
*Z* = 4
Mo *K*α radiation
μ = 0.08 mm^−1^
*T* = 295 K
0.25 × 0.20 × 0.18 mm

#### Data collection

Bruker SMART CCD area-detector diffractometer
Absorption correction: none
7572 measured reflections
2619 independent reflections
1608 reflections with *I* > 2σ(*I*)
*R*_int_ = 0.026

#### Refinement

*R*[*F* ^2^ > 2σ(*F* ^2^)] = 0.044
*wR*(*F* ^2^) = 0.130
*S* = 1.02
2619 reflections
175 parameters
H-atom parameters constrained
Δρ_max_ = 0.13 e Å^−3^
Δρ_min_ = −0.10 e Å^−3^

### 

Data collection: *SMART* (Siemens, 1996 ▶); cell refinement: *SAINT* (Siemens, 1996 ▶); data reduction: *SAINT*; program(s) used to solve structure: *SHELXS97* (Sheldrick, 2008 ▶); program(s) used to refine structure: *SHELXL97* (Sheldrick, 2008 ▶); molecular graphics: *SHELXTL* (Sheldrick, 2008 ▶); software used to prepare material for publication: *SHELXTL*.

## Supplementary Material

Crystal structure: contains datablocks I, global. DOI: 10.1107/S1600536809015244/hg2499sup1.cif

Structure factors: contains datablocks I. DOI: 10.1107/S1600536809015244/hg2499Isup2.hkl

Additional supplementary materials:  crystallographic information; 3D view; checkCIF report

## Figures and Tables

**Table 1:** Hydrogen-bond geometry (Å, °)

*D*—H⋯*A*	*D*—H	H⋯*A*	*D*⋯*A*	*D*—H⋯*A*
C10—H10*A*⋯*Cg*1^i^	0.93	2.90	3.662	139
C15—H15*B*⋯*Cg*2^ii^	0.96	3.20	3.870	128
C16—H16*B*⋯*Cg*1^iii^	0.96	3.17	3.908	135

Symmetry codes: (i) 
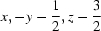
; (ii) 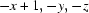; (iii) 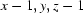. *Cg*1 and *Cg*2 are the centroids of the pyridine and phenyl rings, respectively.

**Fig. 1.**
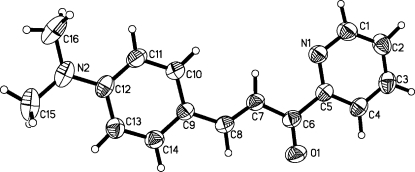
The molecular structure of (I), with atom labels and 30% probability displacement ellipsoids for non-H atoms.

**Fig. 2.**
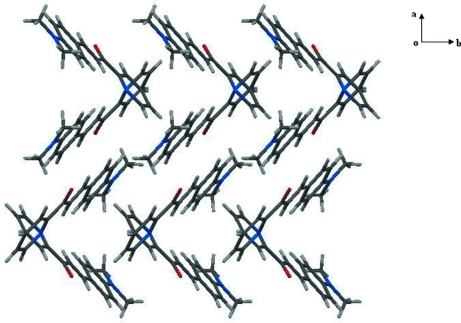
The packing of (I), viewed down the c axis.

## supplementary crystallographic information

### Comment

As an intermediate in the biosynthetic pathway of flavonoids, isoflavonoids, and aurone, chalcones have been shown to display a diverse array of phamacological activities, among which are antifungal, antibacterial, antiprotozoal, anti-inflammatory, antitumor, antimalarial, and anti-HIV activities (Zhao *et al.*, 2007; Fichou *et al.*, 1988; Butcher *et al.*, 2007). In addition, chalcone derivatives are noticeable materials for their excellent blue light transmittance and good crystallizability (Sarojini *et al.*, 2006). In order to research this kind of complex, we synthesis the title compound (I) and report its crystal structure (Fig. 1).

In the title molecule, C_16_H_16_N_2_O, the pyridine ring and the atoms C6,C7,O1 are coplaner (p1), with the largest deviation of 0.045Å for O1. The dihedral angle between p1 and phenyl ring is 2.79 (2)°.

It is interesting to note that the molecular structure is stabilized by intermolecular C—H···π interactions and C—H···N intramolecular interactions (Table 1) [C_g_(1) and C_g_(2) refer to pyridine and phenyl ring, respectively].

### Experimental

5 ml of 10% KOH solution was added to solution of 2-acetylpyridine (1.21 g, 0.01 mol) and 4-(dimethylamino)benzaldehyde (1.49 g, 0.01 mol) in 30 ml ethanol. The solution was stirred for 10 h and filtered. The product obtained was crystallized from acetone/toluene (1:1).

### Refinement

All H atoms were placed in calculated positions, with C—H=0.93–0.96 Å, and included in the final cycles of refinement using a riding model, with *U*_iso_(H)=1.2–1.5 times *U*_eq_(C).

### Crystal data

**Table:**

C_16_H_16_N_2_O	*F*(000) = 536
*M**_r_* = 252.31	*D*_x_ = 1.187 Mg m^−^^3^
Monoclinic, *P*2_1_/*c*	Mo *K*α radiation, λ = 0.71073 Å
Hall symbol: -p 2y bc	Cell parameters from 1586 reflections
*a* = 8.1553 (4) Å	θ = 2.0–25.4°
*b* = 17.4543 (12) Å	µ = 0.08 mm^−^^1^
*c* = 12.1087 (5) Å	*T* = 295 K
β = 125.032 (5)°	Block, red
*V* = 1411.35 (16) Å^3^	0.25 × 0.20 × 0.18 mm
*Z* = 4	

### Data collection

**Table:**

Bruker SMART CCD area-detector diffractometer	1608 reflections with *I* > 2σ(*I*)
Radiation source: fine-focus sealed tube	*R*_int_ = 0.026
graphite	θ_max_ = 25.5°, θ_min_ = 2.3°
φ and ω scans	*h* = −6→9
7572 measured reflections	*k* = −21→20
2619 independent reflections	*l* = −14→14

### Refinement

**Table:**

Refinement on *F*^2^	Secondary atom site location: difference Fourier map
Least-squares matrix: full	Hydrogen site location: inferred from neighbouring sites
*R*[*F*^2^ > 2σ(*F*^2^)] = 0.044	H-atom parameters constrained
*wR*(*F*^2^) = 0.130	*w* = 1/[σ^2^(*F*_o_^2^) + (0.054*P*)^2^ + 0.1706*P*] where *P* = (*F*_o_^2^ + 2*F*_c_^2^)/3
*S* = 1.02	(Δ/σ)_max_ < 0.001
2619 reflections	Δρ_max_ = 0.13 e Å^−^^3^
175 parameters	Δρ_min_ = −0.10 e Å^−^^3^
0 restraints	Extinction correction: *SHELXL97* (Sheldrick, 2008), Fc^*^=kFc[1+0.001xFc^2^λ^3^/sin(2θ)]^-1/4^
Primary atom site location: structure-invariant direct methods	Extinction coefficient: 0.025 (3)

### Special details

**Table:**

Geometry. All e.s.d.'s (except the e.s.d. in the dihedral angle between two l.s. planes) are estimated using the full covariance matrix. The cell e.s.d.'s are taken into account individually in the estimation of e.s.d.'s in distances, angles and torsion angles; correlations between e.s.d.'s in cell parameters are only used when they are defined by crystal symmetry. An approximate (isotropic) treatment of cell e.s.d.'s is used for estimating e.s.d.'s involving l.s. planes.
Refinement. Refinement of *F*^2^ against ALL reflections. The weighted *R*-factor *wR* and goodness of fit *S* are based on *F*^2^, conventional *R*-factors *R* are based on *F*, with *F* set to zero for negative *F*^2^. The threshold expression of *F*^2^ > σ(*F*^2^) is used only for calculating *R*-factors(gt) *etc*. and is not relevant to the choice of reflections for refinement. *R*-factors based on *F*^2^ are statistically about twice as large as those based on *F*, and *R*- factors based on ALL data will be even larger.

### Fractional atomic coordinates and isotropic or equivalent isotropic displacement parameters (Å^2^)

**Table:**

	*x*	*y*	*z*	*U*_iso_*/*U*_eq_	
O1	0.3917 (2)	0.59831 (8)	−0.12014 (13)	0.0974 (5)	
N1	0.1005 (2)	0.75477 (9)	−0.13939 (15)	0.0741 (4)	
N2	0.7635 (3)	0.56873 (11)	0.70210 (17)	0.0942 (6)	
C1	−0.0344 (3)	0.80574 (12)	−0.2313 (2)	0.0888 (6)	
H1A	−0.0832	0.8425	−0.2015	0.107*	
C2	−0.1059 (3)	0.80743 (13)	−0.3672 (2)	0.0873 (6)	
H2A	−0.1993	0.8438	−0.4256	0.105*	
C3	−0.0344 (3)	0.75393 (13)	−0.41174 (19)	0.0831 (6)	
H3A	−0.0778	0.7532	−0.5015	0.100*	
C4	0.1047 (3)	0.70057 (11)	−0.32047 (18)	0.0717 (5)	
H4A	0.1552	0.6637	−0.3489	0.086*	
C5	0.1685 (2)	0.70242 (9)	−0.18518 (17)	0.0606 (4)	
C6	0.3188 (3)	0.64457 (10)	−0.08317 (17)	0.0665 (5)	
C7	0.3733 (3)	0.64595 (10)	0.05562 (17)	0.0662 (5)	
H7A	0.3199	0.6844	0.0793	0.079*	
C8	0.4975 (3)	0.59389 (9)	0.15074 (17)	0.0644 (5)	
H8A	0.5468	0.5563	0.1227	0.077*	
C9	0.5646 (2)	0.58887 (9)	0.29150 (16)	0.0598 (4)	
C10	0.4985 (3)	0.63890 (10)	0.34847 (18)	0.0723 (5)	
H10A	0.4098	0.6779	0.2955	0.087*	
C11	0.5612 (3)	0.63226 (11)	0.48151 (19)	0.0784 (6)	
H11A	0.5121	0.6666	0.5146	0.094*	
C12	0.6978 (3)	0.57483 (11)	0.56871 (18)	0.0704 (5)	
C13	0.7654 (3)	0.52452 (11)	0.51256 (18)	0.0728 (5)	
H13A	0.8554	0.4859	0.5656	0.087*	
C14	0.6996 (3)	0.53165 (10)	0.37868 (18)	0.0680 (5)	
H14A	0.7470	0.4970	0.3449	0.082*	
C15	0.9044 (4)	0.50919 (15)	0.7917 (2)	0.1197 (9)	
H15A	1.0236	0.5121	0.7937	0.180*	
H15B	0.8437	0.4597	0.7591	0.180*	
H15C	0.9381	0.5167	0.8811	0.180*	
C16	0.6813 (4)	0.61785 (15)	0.7560 (2)	0.1247 (10)	
H16A	0.5384	0.6120	0.7031	0.187*	
H16B	0.7140	0.6703	0.7528	0.187*	
H16C	0.7371	0.6037	0.8478	0.187*	

### Atomic displacement parameters (Å^2^)

**Table:**

	*U*^11^	*U*^22^	*U*^33^	*U*^12^	*U*^13^	*U*^23^
O1	0.1348 (12)	0.0967 (10)	0.0907 (10)	0.0326 (9)	0.0822 (10)	0.0081 (8)
N1	0.0852 (11)	0.0825 (10)	0.0739 (10)	0.0124 (9)	0.0569 (9)	0.0096 (8)
N2	0.1095 (14)	0.1125 (14)	0.0666 (11)	−0.0230 (11)	0.0540 (10)	−0.0111 (10)
C1	0.1010 (15)	0.0973 (15)	0.0916 (14)	0.0264 (13)	0.0690 (13)	0.0204 (12)
C2	0.0818 (14)	0.1094 (16)	0.0779 (13)	0.0158 (12)	0.0500 (12)	0.0254 (12)
C3	0.0809 (13)	0.1099 (16)	0.0624 (11)	−0.0060 (12)	0.0433 (11)	0.0071 (11)
C4	0.0804 (13)	0.0803 (12)	0.0680 (12)	−0.0099 (10)	0.0506 (10)	−0.0070 (10)
C5	0.0655 (10)	0.0652 (10)	0.0655 (10)	−0.0091 (9)	0.0459 (9)	−0.0026 (8)
C6	0.0782 (12)	0.0663 (10)	0.0724 (11)	−0.0016 (9)	0.0534 (10)	−0.0032 (9)
C7	0.0756 (11)	0.0681 (11)	0.0684 (11)	0.0040 (9)	0.0491 (10)	−0.0014 (9)
C8	0.0711 (11)	0.0635 (10)	0.0718 (11)	−0.0008 (9)	0.0488 (10)	−0.0052 (9)
C9	0.0618 (10)	0.0621 (10)	0.0624 (10)	−0.0032 (8)	0.0398 (9)	−0.0043 (8)
C10	0.0753 (12)	0.0755 (12)	0.0694 (12)	0.0078 (9)	0.0434 (10)	−0.0048 (9)
C11	0.0841 (13)	0.0875 (13)	0.0743 (13)	−0.0014 (11)	0.0517 (11)	−0.0168 (11)
C12	0.0712 (12)	0.0834 (13)	0.0610 (11)	−0.0225 (10)	0.0404 (10)	−0.0124 (9)
C13	0.0752 (12)	0.0774 (12)	0.0690 (12)	0.0005 (10)	0.0431 (10)	0.0051 (9)
C14	0.0734 (12)	0.0678 (11)	0.0743 (12)	0.0026 (9)	0.0492 (10)	−0.0012 (9)
C15	0.122 (2)	0.150 (2)	0.0671 (14)	−0.0307 (18)	0.0424 (14)	0.0099 (15)
C16	0.170 (2)	0.141 (2)	0.0992 (17)	−0.0506 (19)	0.0988 (18)	−0.0452 (16)

### Geometric parameters (Å, °)

**Table:**

O1—C6	1.2293 (19)	C8—C9	1.463 (2)
N1—C5	1.343 (2)	C8—H8A	0.9300
N1—C1	1.355 (2)	C9—C10	1.398 (2)
N2—C12	1.383 (2)	C9—C14	1.411 (2)
N2—C16	1.454 (3)	C10—C11	1.387 (2)
N2—C15	1.465 (3)	C10—H10A	0.9300
C1—C2	1.396 (3)	C11—C12	1.418 (3)
C1—H1A	0.9300	C11—H11A	0.9300
C2—C3	1.365 (3)	C12—C13	1.403 (2)
C2—H2A	0.9300	C13—C14	1.390 (2)
C3—C4	1.391 (3)	C13—H13A	0.9300
C3—H3A	0.9300	C14—H14A	0.9300
C4—C5	1.406 (2)	C15—H15A	0.9600
C4—H4A	0.9300	C15—H15B	0.9600
C5—C6	1.520 (2)	C15—H15C	0.9600
C6—C7	1.472 (2)	C16—H16A	0.9600
C7—C8	1.356 (2)	C16—H16B	0.9600
C7—H7A	0.9300	C16—H16C	0.9600
			
C5—N1—C1	116.17 (16)	C10—C9—C8	123.02 (16)
C12—N2—C16	120.7 (2)	C14—C9—C8	121.56 (15)
C12—N2—C15	122.19 (19)	C11—C10—C9	122.09 (17)
C16—N2—C15	116.96 (19)	C11—C10—H10A	119.0
N1—C1—C2	125.10 (19)	C9—C10—H10A	119.0
N1—C1—H1A	117.4	C10—C11—C12	122.16 (17)
C2—C1—H1A	117.4	C10—C11—H11A	118.9
C3—C2—C1	117.86 (19)	C12—C11—H11A	118.9
C3—C2—H2A	121.1	N2—C12—C13	121.42 (19)
C1—C2—H2A	121.1	N2—C12—C11	122.41 (18)
C2—C3—C4	118.83 (18)	C13—C12—C11	116.17 (16)
C2—C3—H3A	120.6	C14—C13—C12	120.88 (18)
C4—C3—H3A	120.6	C14—C13—H13A	119.6
C3—C4—C5	119.88 (17)	C12—C13—H13A	119.6
C3—C4—H4A	120.1	C13—C14—C9	123.30 (16)
C5—C4—H4A	120.1	C13—C14—H14A	118.4
N1—C5—C4	122.16 (17)	C9—C14—H14A	118.4
N1—C5—C6	116.63 (15)	N2—C15—H15A	109.5
C4—C5—C6	121.22 (15)	N2—C15—H15B	109.5
O1—C6—C7	122.33 (17)	H15A—C15—H15B	109.5
O1—C6—C5	118.26 (15)	N2—C15—H15C	109.5
C7—C6—C5	119.41 (15)	H15A—C15—H15C	109.5
C8—C7—C6	123.19 (16)	H15B—C15—H15C	109.5
C8—C7—H7A	118.4	N2—C16—H16A	109.5
C6—C7—H7A	118.4	N2—C16—H16B	109.5
C7—C8—C9	128.81 (16)	H16A—C16—H16B	109.5
C7—C8—H8A	115.6	N2—C16—H16C	109.5
C9—C8—H8A	115.6	H16A—C16—H16C	109.5
C10—C9—C14	115.41 (15)	H16B—C16—H16C	109.5

### Hydrogen-bond geometry (Å, °)

**Table:**

*D*—H···*A*	*D*—H	H···*A*	*D*···*A*	*D*—H···*A*
C7—H7A···N1	0.93	2.51	2.846 (2)	102
C8—H8A···O1	0.93	2.55	2.873 (2)	101
C10—H10A···Cg1^i^	0.93	2.90	3.662	140
C15—H15B···Cg2^ii^	0.96	3.20	3.870	128
C16—H16B···Cg1^iii^	0.96	3.17	3.908	135

Symmetry codes: (i) *x*, −*y*−1/2, *z*−3/2; (ii) −*x*+1, −*y*, −*z*; (iii) *x*−1, *y*, *z*−1.
